# Supplementary material for: Characterisation of a touchscreen-based task for assessing cognitive judgement bias in mice: a new translational tool for affective state disorder drug screening
Source: Psychopharmacology (Berl). 2025 Nov 11;243(7):1615–29. doi: 10.1007/s00213-025-06909-5 (PMC13433727; doi:10.1007/s00213-025-06909-5)
Supplement: Supplementary file 1 [file 213_2025_6909_MOESM1_ESM.docx]

**Characterisation of a touchscreen-based task for assessing cognitive judgement bias in mice: a new translational tool for affective state disorder drug screening.**

Laura Lopez-Cruz ^1+^, Benjamin U Phillips ^1^, Lisa M Saksida ^3,4*^, Christopher J Heath ^2*^, Timothy J Bussey ^3,4*^

1. Department of Psychology and MRC/Wellcome Trust Behavioural and Clinical Neuroscience Institute, University of Cambridge, Downing Street, Cambridge, CB2 3EB, UK.

2. School of Life, Health and Chemical Sciences, The Open University, Walton Hall, Milton Keynes, MK7 6AA, UK.

3. Robarts Research Institute & Department of Physiology and Pharmacology, Schulich School of Medicine & Dentistry, Western University, London, ON, N6A 5C1, Canada.

4. Department of Physiology and Pharmacology, Schulich School of Medicine and Dentistry, Western University, London, ON, N6A 5C1, Canada

**Corresponding author**: Laura Lopez-Cruz ([laura.lopez-cruz@open.ac.uk](mailto:laura.lopez-cruz@open.ac.uk))

+Present address: School of Life, Health and Chemical Sciences, The Open University, Walton Hall, Milton Keynes, MK7 6AA, UK.

***Senior authors**

**Tetrabenazine at low doses did not have effects on CJB task performance.**

Two-way ANOVA indicated a main effect of stimulus type on the proportion of Responses observed under TBZ (F(3.437, 96.24) = 114.0, p<0.0001, ηp^2^=0.8028 ) but no main effect of treatment (F (1, 28) = 0.014, p=0.9152, ηp^2^=0.0002 ) or a treatment x stimulus type interaction (F(5, 140) = 1.348, p=0.2477, ηp^2^ =0.0459) was observed (**Fig. S1 a).** Concerning latency to respond, a main effect of stimulus type was detected (F (2.610, 69.43) = 11.19, p<0.0001, ηp^2^=0.2960) in the absence of a main effect of treatment (F (1, 28) = 1,835, p=0.1863, ηp^2^=0.0615) and a treatment x stimulus type interaction (F (5, 133) = 0.7301, p=0.6021, ηp^2^=0.0206) **(Fig. S1 b**).

**Fig. S1 Effects of low doses of TBZ on CJB task primary outcome measures.** Proportion (P) of Responses (**a**) and Latency to respond (**b**) *Data shown as mean (± SEM)*

**Other variables:**

| Drug/Dose (mg/kg) | d’ | c | Blank touches | Correction  trials (P) |
| --- | --- | --- | --- | --- |
| T BZ  0  1.5  3.0  F, p, ηp^2^ | 2.29±0.14  2.57±0.12  2.36±0.15  F (1.77, 24.77)=1.63, p=0.22, ηp^2^=0.10 | 0.06±0.11  -0.05±0.10  0.05±0.10  F (1.64, 24.67)=0.77, p=0.45, ηp^2^=0.05 | 0.06±0.01  0.05±0.01  0.05±0.01  F (1.74, 24.40)=1.74 p=0.20, ηp^2^=0.11 | 0.23±0.09  0.22±0.06  0.14±0.04  F (1.36, 19.07)=0.48, p=0.56, ηp^2^=0.03 |

**Table S1.** Effects of TBZ at low doses on d´, c, blank touches, and proportion of correction trials

**SB 242084 at low doses did not have effects on CJB task performance.**

Two-way ANOVA indicated a main effect of stimulus type on the proportion of Responses observed under SB 242084 (F(4.101, 172.2) = 115.4, p<0.0001, ηp^2^ =0.7331) but no main effect of SB 242084 (F(2, 42) = 0.892, p=0.418, ηp^2^=0.0308) or a treatment x stimulus type interaction (F (10, 210) = 0.448, p=0.959, ηp^2^ =0.0208) was observed (**Fig. S2 a).** For latency to respond, a main effects of stimulus type was detected (F (3.481, 48.73) = 18.10, p<0.0001, ηp^2^=0.5638) in the absence of a main effect of treatment (F (1.423, 19.93) = 0.711, p=0.4573, ηp^2^=0.0483) and a treatment x stimulus type interaction (F (4.311, 50.44)= 0.7946, p=0.5425, ηp^2^=0.0635) **(Fig. S2 b**).

**Fig. S2 Effects of low doses of SB 242084 on CJB task primary outcome measures.** Proportion (P) of Responses (**a**) and Latency to respond (**b**) *Data shown as mean (± SEM)*

***Other variables:***

| Drug/Dose (mg/kg) | d’ | c | Blank touches | Correction  trials (P) |
| --- | --- | --- | --- | --- |
| SB 242084  0  0.25  0.50  F, p, ηp^2^ | 1.96±0.143  2.07±0.11  2.15±0.15  F (1.87, 26.18)=0.72, p=0.50, ηp^2^ =0.05 | 0.21±0.09  **0.02 ±0.09***  0.04±0.08  **F (1.82, 25.53)**  **=4.06, p=0.03, ηp^2^ =0.23** | 0.05±0.01  0.06±0.01  0.06±0.01  F (1.51, 21.14)=3.22, p=0.07, ηp^2^=0.19 | 0.20±0.05  0.18±0.05  0.23±0.07  F (1.73, 24.32)=0.19, p=0.79, ηp^2^=0.01 |

**Table S2.** Effects of citalopram, fluoxetine, and SB 242084 on d´, c, blank touches, and proportion of correction trials. *p<0.05, significantly different from vehicle treatment.

***Summary of front and rear IR beam break rates as a measure of general locomotor activity***

None of the drugs and doses used impaired or stimulated locomotion (Table S3).

| Drug/Dose (mg/kg) | Front beam breaks/sec | Rear beam breaks/sec |
| --- | --- | --- |
| Bupropion |  |  |
| 0  5.0  10.0  F, p, ηp^2^ | 0.30±0.02  0.31±0.03  0.37±0.04  F (1.84, 20.24)=3.28, p=0.06, ηp^2^ =0.23 | 0.06±0.01  0.06±0.01  0.07±0.01  F (1.82, 19.96)=0.6122, p=0.54, ηp^2^ =0.05 |
| TBZ |  |  |
| 0  1.5  3.0  F, p, ηp^2^ | 0.26±0.01  0.26±0.02  0.28±0.03  F (1.763 21.16)=0.30  , p=0.71, ηp^2^ =0.02 | 0.06±0.003  0.06±0.004  0.07±0.006  F (1.55, 18.60)=0.57, p=0.53, ηp^2^ =0.05 |
| TBZ |  |  |
| 0  6.0  t (df), p, *d* | 0.22±0.03  0.26±0.03  t(12)=0.3, p>0.05, *d*= 0.25 | 0.063±0.01  0.061±0.001  (12)=0.78, p>0.05 *d*= 0.08 |
| Citalopram |  |  |
| 0  2.5  5.0  10.0  F, p, ηp^2^ | 0.28±0.02  0.30±0.03  0.29±0.02  0.28±0.03  F (1.71, 22.24)=0.34, p=0.68, ηp^2^ =0.03 | 0.07±0.006  0.07±0.004  0.07±0.007  0.06±0.005  F (2.04, 26.53)=0.73, p=0.49, ηp^2^ =0.05 |
| Fluoxetine |  |  |
| 0  2.5  5.0  F, p, ηp^2^ | 0.25±0.03  0.23±0.03  0.22±0.03  F (1.42, 18.48)=0.95  p=0.38, ηp^2^ =0.07 | 0.07±0.004  0.06±0.004  0.06±0.004  F (1.60, 22.34)=1.370, p=0.27 ηp^2^=0.09 |
| SB242084 |  |  |
| 0  0.25  0.50  F, p, ηp^2^ | 0.20±0.03  0.21±0.03  0.26±0.03  F (1.53, 19.94)=3.63  , p=0.055, ηp^2^ =0.23 | 0.07±0.005  0.08±0.005  0.08±0.005  F (1.919, 24.94)=1.713, p=0.20, ηp^2^=0.12 |
| SB242084 |  |  |
| 0  0.5  1.0  F, p, ηp^2^ | 0.32±0.04  0.38±0.03  0.34±0.04  F (1.34, 16.06)=2.38, p=0.14, ηp^2^ =0.17 | 0.07±0.006  0.07±0.008  0.08±0.007  F (1.93, 21.24)=1.09  p=0.35, ηp^2^ =0.09 |

**Table S3.** Effects of bupropion, TBZ, citalopram, fluoxetine, and SB 242084 on front and rear IR beam break rates.
